# Psychometric evaluation of the Chinese version of the Elderly-Constipation Impact Scale: a translation and validation study

**DOI:** 10.1186/s12889-023-16231-4

**Published:** 2023-07-13

**Authors:** Chen Zheng, Zhen Yang, Linghui Kong, Ziyun Gao, Tingting Lu, Huijun Zhang

**Affiliations:** 1 School of Nursing, Jinzhou Medical University, No.40, Section 3, Songpo Road, Linghe District, Liaoning Province Jinzhou City, P.R. China; 2 School of Nursing, China Medical University, 77 Puhe Road, Shenyang New District, Shenyang, Liaoning Province P.R. China

**Keywords:** Elderly, Chronic constipation, Quality of life, Factor analysis, Psychometric evaluation

## Abstract

**Objective:**

The objective of this study was to translate the Elderly-Constipation Impact Scale into Chinese and to examine its reliability and validity in a population of older people suffering from chronic constipation.

**Methods:**

In this study, the scale was paraphrased, back-translated, cross-culturally adapted and pre-experimented using the Brislin double translation-back-translation method to create the initial Chinese version of the Elderly-Constipation Impact Scale. A convenience sampling method was used to select 564 study participants who met the inclusion and exclusion criteria in Liaoning and Shanxi, China, to evaluate the reliability and validity of the scale. General information about the study population was using descriptive statistics; item analysis was used to screen the items of the scale. Content validity, exploratory factor analysis, and validation factor analysis were chosen to validate the scales; internal consistency, spilt-half reliability and retest reliability were used determine the reliability of the measurement scales.

**Results:**

The Chinese version of the Elderly-Constipation Impact Scale contains 7 dimensions and 21 items. The Cronbach's alpha coefficient for the total scale was 0.901 and the range of Cronbach's alpha values for each dimension was 0.707 to 0.918. The split-half reliability of the scale was 0.736 and the retest reliability was 0.763. The exploratory factor analysis showed a KMO value of 0.873 and a Bartlett's spherical test *X*^*2*^ value of 3499.978 (*p* < 0.001). A total of seven common factors were extracted, namely daily activities, treatment satisfaction, lack of control of bodily function, diet restriction, symptom intensity, anxiety and preventive actions, with a cumulative variance contribution of 77.813%. Each item had a loading value > 0.4 on its common factor. In the validation factor analysis, the model fit results were *X*^*2*^ / df = 1.886, GFI = 0.910, AGFI = 0.874, PGFI = 0.654, IFI = 0.955, TLI = 0.942, CFI = 0.954, RMSEA = 0.056 and PNFI = 0.718. The model fit indicators were all within acceptable limits.

**Conclusion:**

The Chinese version of the E-CIS has good reliability and validity in the chronic constipation population of elderly individuals. The results of the questionnaire can effectively and comprehensively reflect the impact of chronic constipation on the quality of life of elderly individuals. It provides a meaningful reference for identifying targets for intervention.

## Introduction

Population ageing is an inevitable consequence of demographic transformation. The global trend is evident and the population, in general, is now an ageing society [1, 2]. Not only is the elderly population large and growing, but the proportion of them suffering from chronic diseases is also on the rise [3]. Chronic diseases are gradually becoming a major threat to the physical health and well-being of elderly people [4–6]. Constipation is one of the common chronic diseases of elderly individuals [7, 8]. It poses a great burden on individuals, families, and society in terms of physical and mental health. Severe chronic constipation not only increases the cost of medical care but also represents a financial burden for providers [9, 10].

Chronic constipation is considered a common disorder of the gastrointestinal tract and is often considered to be a simple, easily treatable condition. Its main symptoms include reduced frequency or difficulty in passing stools, as evidenced by dry stools, difficult or incomplete bowel movements, prolonged bowel movements, and the need to pass stools manually. Age has been widely demonstrated to be a significant predictor of the occurrence and development of chronic constipation among elderly individuals [4, 11]. Compared to other common chronic diseases, chronic constipation significantly reduces the quality of life of patients [6, 12, 13]. Physiologically, as the disease progresses, chronic constipation, if left untreated, may cause or aggravate more serious rectal diseases such as faecal impaction, perforation, anal fissures, enteritis, hemorrhoids, and rectal cancer [12, 14]. Psychologically, because of the high financial burden, prolonged illness, and lack of effective health management, older people with chronic constipation can also suffer from psychiatric disorders such as irritability, anxiety, and depression [15, 16]. In severe cases, this can even lead to depression, somatic disorders, cognitive dysfunction, and even suicidal tendencies [17, 18]. Social support is an important influencing factor. Good social support not only reduces the stigma of the disease but also increases adaptive behaviors and reduces the negative impact of stressful events [16, 19]. It can lead to the adoption of positive strategies to alleviate the physical and psychological symptoms associated with chronic constipation and improve treatment compliance and confidence.

The prevalence of constipation in the elderly population is gradually increasing due to changes in diet and lifestyle as well as psychological and social factors caused by the increasing age of elderly individuals [20, 21]. With the emergence of the medical model, the evaluation of conditions based solely on symptoms is no longer relevant. As chronic constipation is a common physical and psychological condition, quality of life assessment is also becoming increasingly important as a psychosocial indicator for the diagnosis and treatment of chronic constipation and the evaluation of efficacy [10]. The Patient Assessment of Constipation Quality of Life (PAC-QoL) scale is widely used in of adults with chronic constipation but lacks specificity and age sensitivity [22]. In addition, there is some variation in the measurement of other scales, with Szeinbach et al. focusing on studies of patient satisfaction and Neri et al. preferring to measure the severity of somatic symptoms [23, 24]. In contrast, most of the existing constipation-quality-of-life scales have been developed in Western countries and focus on Western cultural habits and lifestyles, lacking reference to age sensitivity and cultural differences for older individuals in Asian countries [25, 26].

The Elderly-Constipation Impact Scale (E-CIS), developed by Professor Patimah, is a scale developed specifically to measure the impact of chronic constipation on the quality of life of elderly patients [25]. The scale was rationally designed based on the background of the high prevalence and long duration of chronic constipation in the elderly population, taking full account of patient' disease control and cognitive level. The questionnaire is simple and easy to complete and has been shown to have good reliability and validity in the Malaysian population. It can be used to measure the quality of life of elderly patients with chronic constipation in other Asian countries. The scale was developed in 2020 and has been used less in other countries and in China, and no studies on its reliability and validity have been reported. The aim of this study was to introduce the Malaysian version of the E-CIS into China, to assess the reliability and validity of the translated scale and to provide a scientific basis for its application in clinical settings.

## Methods

### Study design and participants

This cross-sectional study was conducted from March to September 2022 to assess the impact of chronic constipation on quality of life among elderly individuals. The sample size was established in accordance with the general rules of the factor analysis procedure, which required a minimum of 10 respondents to be recruited for each project, although larger samples were desirable [27]. Therefore, this study used convenience sampling to recruit participants from two locations in China, Jinzhou, Liaoning, and Xinzhou, Shanxi. The survey sites were located in community street offices and interviewers conducted face-to-face interviews with the respondents. Finally, 564 participants were recruited. Inclusion criteria for the study population required participants to be > 60 years of age; have chronic constipation; be conscious and able to communicate normally; and be voluntarily engaged in this study with informed consent. Patients with cognitive impairment, inpatients, patients with severe hearing impairment, and patients with a stoma and gastrointestinal cancer were excluded.

### Measures

#### General information

The questionnaire for demographics in general was developed by the researcher following a review of the literature and group discussions. Respondents were requested to respond to the following five areas: age, gender, education, occupational status, and general health status.

#### Elderly-constipation impact scale questionnaire

The scale was developed by Patimah et al. to measure the extent to which chronic constipation affects older people [25]. The scale included 22 items in 7 dimensions. Each item uses a 5-point Likert scale, from "never or very rarely" to "always" on a scale of 1 to 5. The higher the score, the greater the impact of chronic constipation on the quality of life for elderly individuals. Treatment satisfaction (with four items) was reverse-scored. In the cross-cultural adaptation, the item "I feel interrupted to pray because of the constipation " was removed, resulting in a 7-dimensional, 21-item Chinese version of the Elderly-Constipation Impact Scale. Scores on the scale range from 21 to 105, with higher scores indicating a greater impact of chronic constipation on the quality of life of older people.

### Procedure

#### Scale translation procedure

Our translation work was approved by Professor Patimah and authorization was obtained. The English version of the Elderly-Constipation Impact Scale was translated by the research team in strict accordance with the Brislin double back-translation principle [28]. Firstly, the questionnaire was translated and completed by two bilingual native Chinese speakers. The research team discussed and agreed on the parts that differed significantly from the original. The questionnaire was then back-translated into English by two independent native English speakers who had not been exposed to the source scale and had Chinese language skills. The translated scales were compared and modified group discussion to produce the final back-translated version. Psychologists were invited to culturally adapt the translated scale so that the items would be more in line with Chinese language expressions. The investigators conducted a pre-experiment on 30 study who met the inclusion and exclusion criteria. After completion, participants were asked if there were any semantic expressions that were ambiguous or if any of the content was difficult to understand. Based on the feedback, changes were made to the expression of the scale items. The final version of the Elderly-Constipation Impact Scale was developed by making appropriate adjustments to the wordings of the items according to the participants' feedback.

#### Data collection procedure

After receiving training, the research team used convenience sampling methods and travelled to two separate cities to recruit study participants. The researchers explained the purpose of the questionnaire and the content to the participants using a uniform guideline. Once the questionnaires were completed, they were checked and verified by the research team and then collected. The final survey was completed by 590 elderly participants suffering from chronic constipation, and 564 valid questionnaires were obtained. 30 elderly patients with chronic constipation were asked to complete the post-translation scale again two weeks later.

#### Data analysis procedure

This study was statistically analyzed using SPSS 26.0 and AMOS 26.0. Means, standard deviations and percentages were used to statistically describe the general demographic information of the study population. *P* < 0.05 means that the difference results are statistically significant.

#### Items analysis

Item analysis aimed to determine the discrimination and relevance of the scale, and the results can be used as a basis for item selection or modification. The critical ratio method and the correlation coefficient method are usually used. The critical ratio method ranked the study participants by summing the total scores of the questionnaire items. Those with scores lower than the top 27% of the total score were classified as the high group and those with scores lower than the bottom 27% of the total score were classified as the low group. To determine whether the difference between high and low groupings, significance was set at *p* < 0.05. A critical ratio (CR) > 3.000 indicate that the items were well discriminated [29, 30]. Correlation coefficients were used to evaluate the independence and homogeneity of the items. The higher the correlation between each item and the total score of the scale, the higher the homogeneity. A correlation coefficient of *r* > 0.4 indicated that the items were well correlated [27]. If the Cronbach's alpha coefficient increased after deleting each item, it meant that the item was different from the attributes measured by the other items and should be excluded [31].

#### Reliability analysis

Reliability, an indicator of the accuracy or consistency of a measurement instrument's response to a measurement result, reflects the degree of truthfulness of the characteristic being measured. That is, whether the measurement instrument can measure the subject or variable being measured in a true and consistent manner. The more consistent the results of a test are, the lower the error and the higher the reliability obtained [32, 33]. In this study, reliability was tested using internal consistency and retest reliability [34]. Cronbach's alpha coefficients were calculated for the translated scale and each dimension as a means of evaluating the internal consistency of the scale [35]. Retesting 30 elderly patients with chronic constipation after two weeks using a post-translation scale to measure the stability and consistency of the scale across time. The split-half reliability was obtained by dividing the scale items into two halves and calculating the correlation between the results of these two halves.

#### Validity analysis

Validity is a judgement of how accurately a scale measures the desired content [33]. In this study, validity was tested in terms of both content and construct validity. Content validity refers to the extent to which the items of a research instrument reflect the content to be measured. Experts in the relevant field were invited to assess the content validity index of the questionnaire's items (I-CVI) and the content validity index of the scale (S-CVI) through Delphi expert correspondence [29]. In this study, seven experts were invited to evaluate the scale items using a 4-point Likert scale. Based on its relevance to the theme, each item was placed in one of four categories: no relevance (1 point), weak relevance (2 points), moderate relevance (3 points), and strong relevance (4 points). The items were evaluated by each expert, and the percentage of experts who selected 3 or 4 was calculated. I-CVI = number of experts scoring 1 for each item / total number of experts; S-CVI = average of the content validity of all items. An I-CVI > 0.800 and an S-CVI > 0.900 indicated good content validity of the scale and a high degree of relevance and representativeness of the scale items [36].

The group of 564 cases was split randomly into two groups, one group (*n* = 282) was used for exploratory factor analysis and the other group (*n* = 282) was used for confirmatory factor analysis. We required the following in validity analysis: (1) Kaiser–Meyer–Olkin (KMO) values > 0.6; (2) Bartlett's spherical test for significant difference (*p *< 0.05); (3) No apparent double loading of the title item; (4) Title item load > 0.5; (5) Cumulative variance explained > 50%. The model's fit indicators were tested in the CFA using AMOS to verify that they were within acceptable limits [37].

## Results

### General information

A total of 564 individuals participated in this study, and the mean age of the participants was 70.67 ± 7.06 years. There were 193 males (34.2%) and 371 females (65.8%). More than half of the respondents were retired (69.5%). The total average score of the Chinese E-CIS was between 21 and 105 points. The average score was 77.48 ± 12.82. Other socio-demographic statistics are shown in Table 1.

**Table 1:** General demography data (*n* = 564)

Factors	Group	n	%
Age	60–69	283	50.2
	70–79	191	33.8
	≥ 80	90	16
Sex	Male	193	34.2
	Female	371	65.8
Education level	Primary school and below	301	53.4
	Junior high school	172	30.5
	Senior Secondary and above	91	16.1
Work	Employed	72	12.8
	Retired	392	69.5
	Unemployed	100	17.7
General health status	Poor to very poor	52	9.2
	Moderate	415	73.6
	Good to very good	97	17.2

### Cross-cultural adaptation

Due to linguistic habits and cultural differences, the scale needs to be adapted cross-culturally to suit the specificities of the local culture to ensure that the important content and basic structure to be conveyed by the target scale is equivalent to that of the source scale to make it more applicable to the target population. After obtaining the author's permission, the researcher made cross-cultural adaptations to the original scale. In the Chinese cultural context, most participants adhered to a materialistic ideology, fewer had religious beliefs and most were not devout believers and were reluctant to admit their beliefs in front of the collectors. In the pre-survey, over 90% of the study participants chose "never or very rarely" for the prompt about religious practices. Based on expert opinion and group discussion, "I feel interrupted to pray because of the constipation " was removed. The final result was a 7-dimensional, 21-item Chinese version of the Elderly-Constipation Impact Scale.

### Item analysis

The critical ratio (CR) values for the 21 items on the scale in this study ranged from 11.010 to 19.683, all of which were > 3. The items were positively correlated with the total score (*r* = 0.496 to 0.675,* P* < 0.001). After deleting the items, the scale’s Cronbach 's α coefficient values were 0.893 ~ 0.899, which did not exceed the original scale’s Cronbach 's α coefficient of 0.901. Therefore, each item could be retained. (Table 2).

**Table 2:** Item analysis for Chinese version of the Elderly-Constipation Impact Scale

Item	Critical ratio	Correlation coefficient between item and total score	Cronbach’s Alpha if item delete
1	14.166	0.594	0.896
2	11.010	0.496	0.899
3	13.287	0.526	0.898
4	19.683	0.643	0.894
5	18.643	0.675	0.893
6	16.876	0.617	0.895
7	15.904	0.589	0.896
8	18.595	0.620	0.895
9	14.285	0.628	0.895
10	15.034	0.630	0.895
11	12.732	0.556	0.897
12	12.428	0.580	0.896
13	14.811	0.511	0.899
14	15.044	0.585	0.896
15	12.020	0.533	0.897
16	13.531	0.563	0.897
17	13.465	0.569	0.896
18	14.175	0.567	0.897
19	12.242	0.559	0.897
20	12.047	0.549	0.897
21	12.045	0.544	0.897

### Reliability analysis

The Cronbach 's α coefficient for the translated scale was 0.901, and the Cronbach 's α coefficient for each dimension ranged from 0.707 to 0.918. The split-half reliability was 0.736. Thirty study participants were selected for retesting after two weeks with a retest reliability of 0.763.

### Validity analysis

#### Content validity

The number of experts with a rating of "1" was counted according to the experts' scores. The content validity (I-CVI) at the item level was calculated to be 0.857 to 1, and the content validity (S-CVI) at the scale level was 0.952.

Exploratory Factor Analysis.

**Fig. 1:**
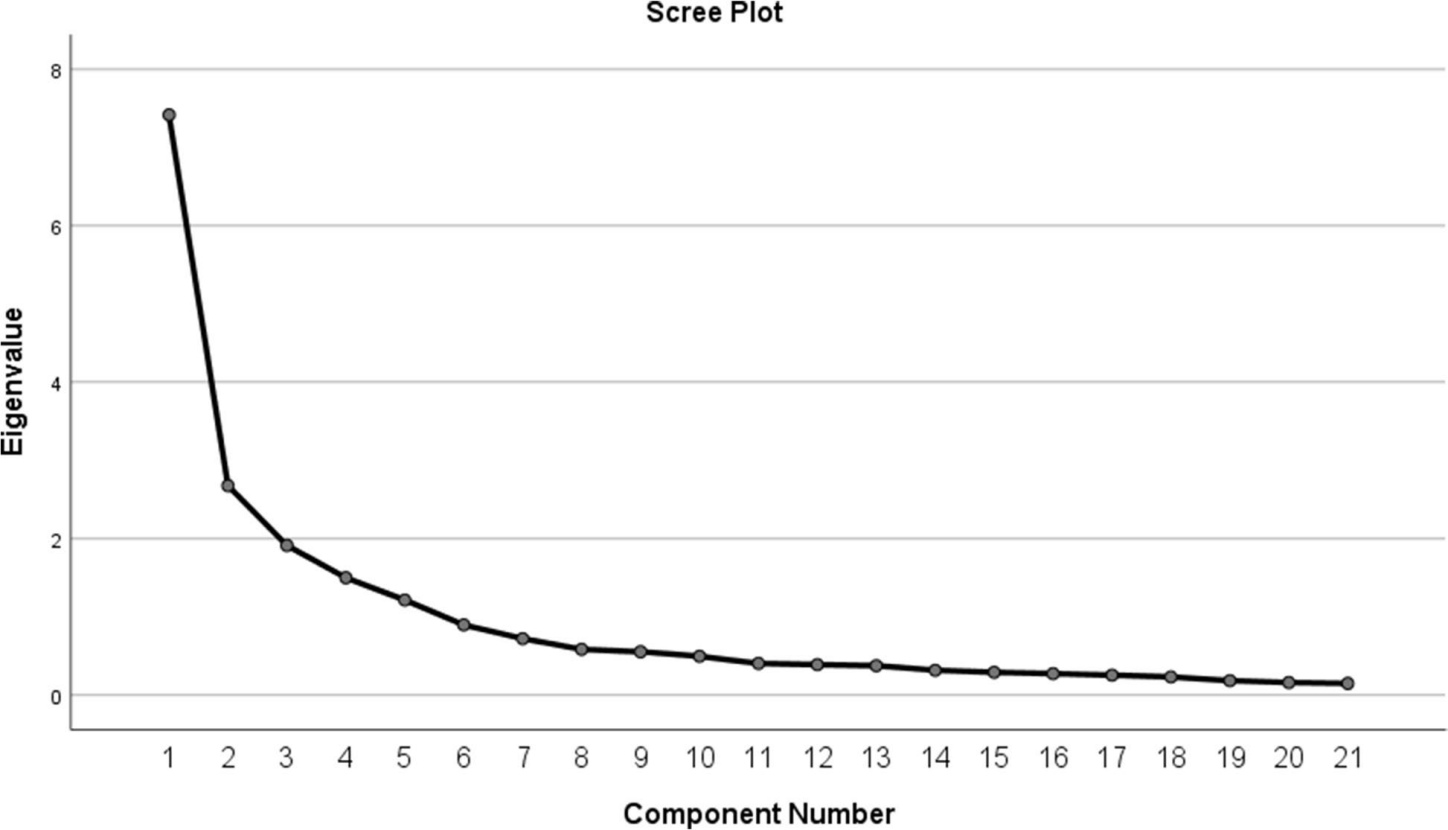
Screen plot of exploratory factor analysis for Chinese version of the Elderly-Constipation Impact

In this study, the KMO value was 0.873 and the chi-squared value of the Bartlett's sphericity test was 3499.978 (*p* < 0.001), indicating a good correlation between the variables and suitability for validated factor analysis. Factor loadings are shown in Table 3. In addition, the 7 factors explained 77.813% of the variance, as confirmed by the scree plot. (Fig. 1).

**Table 3:** Factor loadings of exploratory factor analysis for Chinese version of the Elderly-Constipation Impact Scale

Factor	Factor 1	Factor 2	Factor 3	Factor 4	Factor 5	Factor 6	Factor 7
1	-	-	-	0.831	-	-	-
2	-	-	-	0.805	-	-	-
3	-	-	-	0.777	-	-	-
4	0.885	-	-	-	-	-	-
5	0.868	-	-	-	-	-	-
6	0.846	-	-	-	-	-	-
7	0.783	-	-	-	-	-	-
8	0.598	-	-	-	-	-	-
9	-	-	-	-	-	-	0.754
10	-	-	-	-	-	-	0.728
11	-	-		-	-	0.791	-
12	-	-	-	-	-	0.779	-
13	-	-	-	-	0.792	-	-
14	-	-	-	-	0.791	-	-
15	-	0.861	-	-	-	-	-
16	-	0.836	-	-	-	-	-
17	-	0.809	-	-	-	-	-
18	-	0.696	-	-	-	-	-
19	-	-	0.891	-	-	-	-
20	-	-	0.890	-	-	-	-
21	-	-	0.888	-	-	-	-

### Confirmatory factor analysis

**Fig. 2:**
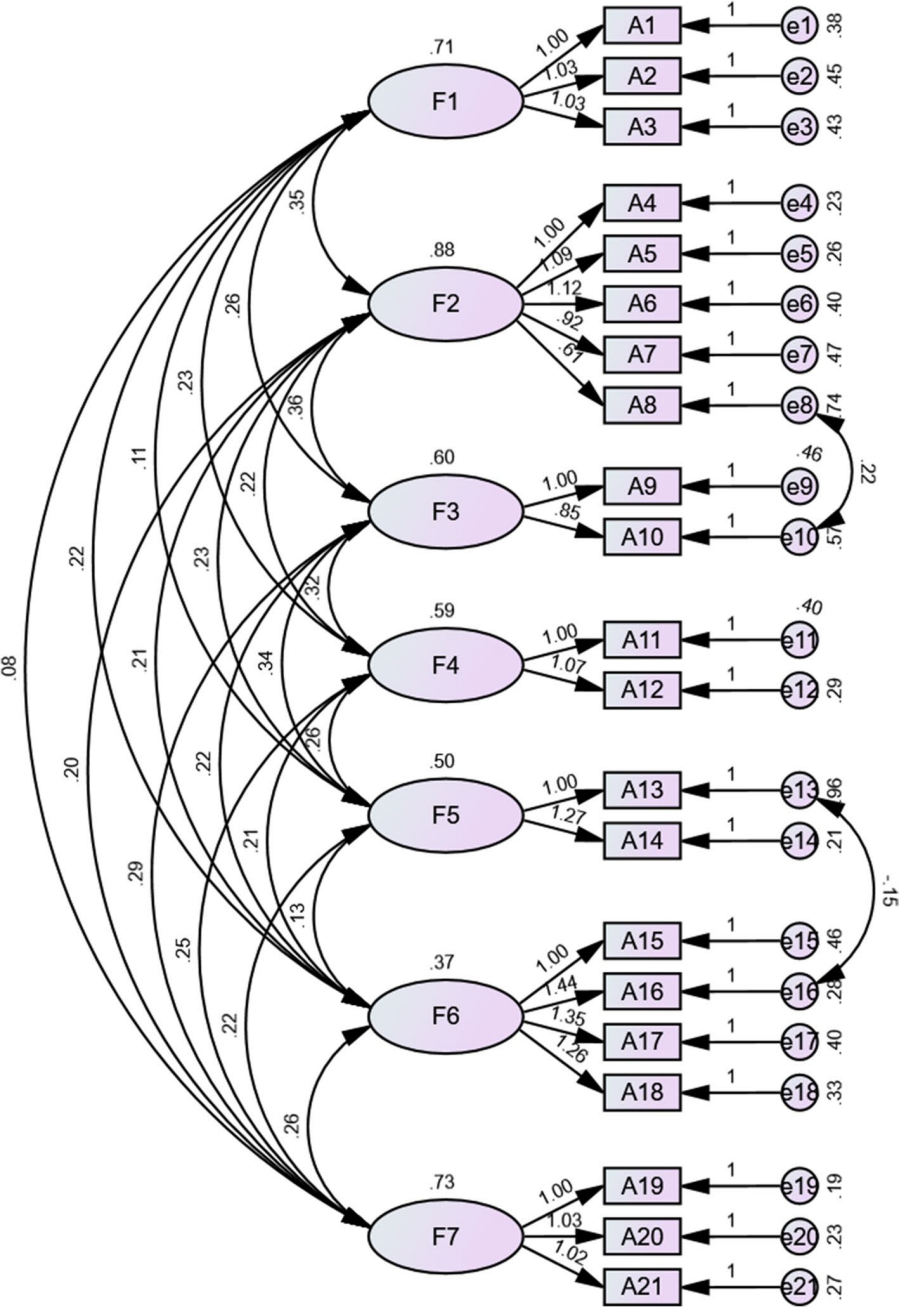
Standardized seven-factor structural model of the Elderly-Constipation Impact Scale

The validated factor analysis was carried out using AMOS software based on a 7-factor structural model, following the maximum likelihood method. In accordance with the Modification Index (MI), the initial model was modified by adding two residual paths: e8 vs e10 and e13 vs e16. The results of the analysis are shown in Fig. 2. The results of each fitted indicator after correction showed that the *X*^2^ / df was 1.886, the GFI was 0.910, the AGFI was 0.874, the PGFI was 0.654, and the IFI was 0.955. The TLI was 0.942, the RMSEA was 0.056, the PNFI was 0.718, and the CFI was 0.954. The analysis of the confirmatory factors was within the reference range for each of the fitted indicators.

## Discussion

With the development of the global economy and continuous advances in social medicine, ageing is increasing worldwide. Chronic constipation is increasingly becoming a public health problem affecting the healthy life expectancy and quality of life of the elderly population. Because of its high prevalence, long duration and persistence, attention is gradually turning to the evaluation of the quality of life of patients with chronic constipation [6, 38, 39]. The measurement tools widely used in China are generic scales. The lack of age specificity of these scales results in a low sensitivity and makes it more difficult to reflect the degree of change in quality of life of aging patients with constipation. The use of scientifically validated tools to assess the health-related quality of life of elderly patients with chronic constipation is a prerequisite for effective intervention. In China, there is no assessment tool that is specific to the health-related quality of life of elderly patients with chronic constipation. The Elderly-Constipation Impact Scale (E-CIS), developed by Professor Patimah, fills this gap [25]. The E-CIS assesses seven areas: daily activities, treatment satisfaction, lack of control of bodily function, diet restriction, symptom intensity, anxiety and preventive actions, providing a valid and comprehensive picture of the impact of chronic constipation on individuals' quality of life. In this study, the E-CIS scale was translated into Chinese for the first time after rigorous translation and reliability testing, and its psychometric characteristics in the elderly constipation population were verified by reliability and validity tests. The results showed that chronic constipation had a significant impact on the quality of life of older Chinese people, which was associated with patients not receiving timely individualized treatment and poor awareness of the associated disorders, in line with the Erichse´ n, Meinds et al. study [40, 41]. Furthermore, the Chinese version of the E-CIS in this study demonstrated appropriate reliability and validity which could be used to assess the impact of chronic constipation on patients' quality of life and to identify the intrinsic needs of patients to enable them to improve. It provided a theoretical basis for clinical practitioners to develop interventions for elderly patients with constipation and to scientifically manage patients' related perceptions and daily behaviors.

The study followed the Brislin translation principles and experts in the relevant fields were invited to debug the first post-translation draft in accordance with the relevant guidelines and Chinese expression conventions [28]. The scale has a total of 21 items, which is a relatively small number of items. Item analysis indicated a high degree of discrimination between the items of the Chinese version of the E-CIS, with each item being too highly correlated with the scale. The removal of each item resulted in a Cronbach's alpha coefficient that did not exceed the original value. This means that all 21 items in the Chinese version of the E-CIS can be retained with good discrimination.

This study examines the reliability of the Chinese version of the E-CIS in terms of internal consistency reliability, retest reliability, and split-half reliability. The results showed that the Cronbach 's α coefficient for the scale was 0.901, which was higher than the results of the Malaysian version, while the stability of the translated scale was found to be at a very favorable level [25]. The Cronbach 's α coefficient for each dimension ranged from 0.707 to 0.918. The split-half reliability was 0.736. The retest reliability after two weeks was 0.763. A retest reliability of > 0.800 is considered high. The results indicate that the reliability of the scale is good.

In this study, the validity of the scale was analyzed and evaluated in terms of both content validity analysis and structural validity analysis. Content validity reflects whether the items meet the measurement purpose and requirements, while construct validity describes the extent to which the theoretical assumptions of the scale match the actual measurement. The I-CVI of the Chinese version of the E-CIS was between 0.857 and 1, and the S-CVI was 0.952, which was higher than the reference value of content validity and had better content validity [36]. In this study, the construct validity of the Chinese version of the E-CIS scale was examined by exploratory factor analysis and confirmatory factor analysis. The Bartlett's spherical test *X*^*2*^ value for the scale was 3499.978 (*p* < 0.001), and the KMO value was > 0.5, making it suitable for factor analysis. The data were subjected to principal component analysis and maximum variance orthogonal rotation, and items with loadings greater than 0.40 within individual factors were selected for inclusion. A total of seven common factors were extracted, explaining 77.813% of the total variance. All items in the component matrix had loadings above 0.4 on the dimension to which they belonged, and no items were deleted [42, 43]. The results of exploratory factor analysis showed that three factors contained only two items. For the two-item factor, reliability can be measured using Spearman-Brown reliability estimates in addition to the Cronbach's alpha coefficient [44]. The higher the reliability, the stronger the correlation between the items. In the Chinese version of E-CIS, the Cronbach's alpha coefficients of the above dimensions ranged from 0.707 to 0.758, and the Spearman-Brown reliability coefficients ranged from 0.711 to 0.758, all of which were > 0.7. This indicated that the internal consistency and stability of these three dimensions were good and acceptable [45]. And the overall fit of the model was good. Therefore, seven dimensions and 21 items were retained in the exploratory factor. The public factor and the items to which it belongs generally conform to the scale development structure and have good structural validity. Meanwhile, in the confirmatory factor analysis, all the fitness indicators of the Chinese version of the E-CIS model were within the acceptable range and the structural validity was good, and some of them were stronger than the fit indicators of the original questionnaire. Overall, the Chinese version of the scale showed optimal validity in elderly patients with chronic constipation [37, 46].

## Limitations

The following limitations exist in this study: although the sample size met the criteria for the study, the sample was limited to a select number of provinces in China. This narrow selection process and weak representativeness, prevent definitive and comprehensive inferences. Future research should expand the scope and sample size of the sample and conduct large multicenter studies across China.

## Conclusion

This study followed a rigorous process of translation, back-translation, cross-cultural adaptation, pre-experimentation, reliability and validity testing. It successfully introduced the Malaysian version of the Elderly-Constipation Impact Scale for the Elderly into China with good psychological properties. In the context of rapid global aging, this study provides a validated tool to assess the impact of chronic constipation on the quality of life of older people, to develop educational programs and interventions for researchers and to lay the foundation for future research related to health promotion in the older population.

## Data Availability

The datasets generated and/or analyzed during the current study are not publicly available to preserve anonymity of the respondents but are available from the corresponding author on reasonable request.
